# Patterns of recurrence after curative D2 resection for gastric cancer: Implications for postoperative radiotherapy

**DOI:** 10.1002/cam4.3085

**Published:** 2020-05-18

**Authors:** Jing Xu, Li Shen, Yongjie Shui, Wei Yu, Qingqu Guo, Risheng Yu, Yulian Wu, Qichun Wei

**Affiliations:** ^1^ Department of Radiation Oncology the Second Affiliated Hospital and Cancer Institute (National Ministry of Education Key Laboratory of Cancer Prevention and Intervention) Zhejiang University School of Medicine Hangzhou P.R. China; ^2^ Department of Surgery the Second Affiliated Hospital Zhejiang University School of Medicine Hangzhou P.R. China; ^3^ Department of Radiology the Second Affiliated Hospital Zhejiang University School of Medicine Hangzhou P.R. China

**Keywords:** gastric cancer, radical surgery, radiotherapy, recurrence patterns

## Abstract

**Background:**

High‐quality randomized controlled trials have demonstrated the benefit of radiotherapy (RT) in patients with radical resected gastric cancer (GC), however, utilization rates of postoperative RT remain remarkably low. Patterns, incidences, and time of recurrence provide biological bases for clinical monitoring of GC patients and guiding potential complementary therapies. Thus, the aim of this study is to understand the location of locoregional recurrence which may allow individualized RT strategies and minimize radiation‐related toxic effects.

**Methods:**

A relatively large sample of GC patients in a single institution who had undergone curative D2 resection was retrospectively reviewed and the relevant recurrence patterns were illustrated. Independent recurrence‐related risk factors were analyzed by logistic regression analysis. New logistic regression models were further developed to predict the probability of recurrence.

**Results:**

Overall, among 776 GC patients who had continuous and complete follow‐up data, 300 cases relapsed after curative resection. Lymphovascular invasion, lymph node metastases, and tumor stage were indicators for early recurrence. Peritoneal, regional, local, and distant recurrence initially occurred in 51 (6.6%), 151 (19.4%), 56 (7.2%), and 164 (21.1%) patients, respectively. Among patients with regional recurrence, the most common sites were lymph node stations 16a2, 8, 12, 16b1, and 9. Remnant stomach recurrence was not so prominent that it seemed reasonable to be excluded from an irradiation field for patients with negative surgical/pathologic margins.

**Conclusions:**

For GC patients who underwent radical D2 resection, distant and regional recurrences were still common. Besides, optimizing regional control of lymph nodes outside the D2 dissected area was crucial for rational design of the RT field. Furthermore, the new logistic regression models might act as useful tools to evaluate recurrence risk and determine which patients should receive postoperative chemoradiotherapy.

## Graphical abstract

Recurrence patterns of a relatively large sample of gastric cancer patients were retrospectively illustrated. All detailed information was critical for selecting appropriate therapeutic modalities and delineating individualized radiotherapy target coverage.

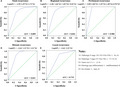

## 1 INTRODUCTION

Gastric cancer (GC) is the second leading cause of cancer‐related mortality worldwide.^1^ Despite improvements in the surgical treatment of GC, 5‐year overall survival rate is only 24.5% in Europe ^2^ and 40%‐60% in Asia.^3^, ^4^ Recurrence after curative resection of GC remains common, and long‐term prognosis of patients is still unsatisfactory due to the high incidence of recurrence. For GC patients receiving complete resections, 79% may suffer recurrences within 2 years, and the median time to death after recurrence is only half a year.^5^ Therefore, appropriate adjuvant therapies should be utilized for preventing recurrence disease in GC. Publication of the Intergroup 0116 trial revolutionized the treatment of resected GC, with improved survival in patients receiving adjuvant chemoradiotherapy (CRT) as compared with surgical resection alone.^6^ Furthermore, the addition of adjuvant radiotherapy (RT) to chemotherapy was associated with a significant overall survival in a National Cancer Data Base analysis for patients with resected GC.^7^

However, despite high‐quality randomized controlled trial evidence demonstrating the benefit of postoperative RT for GC patients, utilization rates of adjuvant RT remained remarkably low.^8^ It was demonstrated that ≥ 85% of the cohort who had stage Ib‐IVM0 disease could be eligible for adjuvant RT, nevertheless, only 30.4% of the patients received adjuvant RT.^9^ The volume of irradiated tissue in clinical trials was large, encompassing the residual stomach, anastomosis site, gastric bed, and regional lymphatics.^10^ Hence, even with technical advancment in incorporating three‐dimensional conformal RT, intesity‐modulated RT, and image‐guided RT, wide application of RT was still restricted due to the dose‐limiting toxicity in GC patients.^11^

Reliable descriptions of the recurrence patterns have important implications for guiding potential complementary therapies. Meanwhile, specific location of locoregional recurrence should be illustrated to define accurate radiation field and minimize radiation‐related toxicity. The aims of this study were to thoroughly understand the incidences, patterns, and time of recurrence and to explore the related risk factors to guide target delineation.

## 2 METHODS

### 2.1 Patients

From January 2010 to December 2013, 1511 consecutive GC patients from a single institution who had undergone curative resection for primary GC at the department of surgery in the Second Affiliated Hospital, Zhejiang University School of Medicine, were retrospectively analyzed. The study was approved by the institutional review board. All patients had histologically confirmed gastric adenocarcinoma at the time of resection. In order to eliminate the possible influence of preoperative therapy to the recurrence patterns, patients who received preoperative CRT or RT or chemotherapy were excluded. Prior to 2013, very few GC patients received postoperative RT in our institution. Hence, GC patients who received postoperative RT were excluded. The operative procedure, either radical total or subtotal gastrectomy, was undertaken depending on tumor location and macroscopic type. All patients underwent potentially curative resection and D2 lymphadenectomy. D2 lymphadenectomy involved complete dissection of lymph nodes (LNs) in station 1‐12.^12^ Adjuvant chemotherapy was routinely recommended for stage II or III GC patients, except for those with a poor performance status or severe complications. For the adjuvant chemotherapy regimens, 5‐fluorouracil‐based regimens were most common, including FOLFOX, XELOX, FOLFIRI, fluoropyrimidines, SOX, etc. Cancers were staged according to the eighth edition of the American Joint Committee on Cancer/Union for International Cancer Control tumor node metastasis (TNM) classification.^13^ Among the 1511 patients, 177 patients had peritoneal or hepatic metastasis at the time of surgery, 34 patients received neoadjuvant chemotherapy, 2 patients received postoperative RT, and 29 patients had double primary malignancy. Finally, the remaining 1269 patients were included for further analysis.

Clinicopathologic information and tumor characteristics were collected, including age, gender, primary tumor location, histology, maximum diameter, tumor stage, perineural, and lymphovascular invasion. Therapeutic data were also analyzed retrospectively, including type of resection and reconstruction, as well as number of dissected LNs and positive lymph node ratio.

### 2.2 Follow‐up

After gastrectomy, patients were followed up regularly. It was recommended that follow‐up intervals were every 3 months for the first 2 years, 6 months for the next 3 years, and annually examinations thereafter. Regular schedule of evaluation was carried out with the medical history, physical examination, laboratory tests, chest radiography, abdominopelvic ultrasonography, computed tomography of the abdomen (contrast‐enhanced computed tomography with iopromide if no allergy before), gastroscopy, and bone scintigraphy for the detection of suspicious skeletal metastasis. Ultimately, continuous and complete follow‐up data in our hospital were recorded from 776 patients until 31 Jan 2018. The distance from home to hospital was a factor individual considered for reexamination. Meanwhile, where one works and lives is the factor most strongly related to the likelihood of being medically insured. Finally, the other 493 patients chose to be followed up in local medical institutions by local physicians.

### 2.3 Definition of Recurrence

Recurrence diagnosis was based on all available radiographic and histopathologic reports. The length of time to recurrence was defined from the time of surgery to the diagnosis of detectable recurrence. Recurrence patterns were recorded and analyzed as the first recurrence occurred during the entire follow‐up. Actually, a subset of patients suffered only one recurrence pattern during the course of disease. For the remaining patients who experienced two or more sites of recurrences, they were recorded separately according to the recurrence pattern.

Patterns of recurrence were classified as peritoneal (peritoneum, colorectum, and ovary), regional (regional lymph node), local (anastomotic site, gastric bed, and remnant stomach), and distant recurrence. We defined remote lymphatic metastasis such as pelvic nodes, supraclavicular and infraclavicular nodes as distant lymphatic recurrence.

### 2.4 Statistical analysis

All clinicopathological and follow‐up data were stored in a database. Statistically significant differences were compared using the two‐tailed χ2 test and Student's τ test. All statistical analyses were carried out with the SPSS for Windows (SPSS, Chicago, Illinois, USA) program.

The risk variables that related with patterns of recurrence were determined by univariate and multivariate logistic regression analysis. The odds ratio (OR) with the 95% confidence intervals in logistic regression analysis was defined as the ratio of odds that an event would occur to the probability that it would not occur. Additionally, the predicted model of recurrence was also calculated according to the results of logistic regression analysis. The performance of the model was studied with respect to discrimination. Discriminative ability was determined with the concordance c‐statistic. The interpretation of the c‐statistic was comparable to the interpretation of the Area Under the Curve (AUC) of the Receiver Operator Curve (ROC) and could be applied to ordinal regression models. The maximum value of the c‐statistic was 1.0, which indicated a perfect discrimination. The values of Logit(P) were derived by multivariate logistic regression analysis. The probability of recurrence was estimated as P, and we could further obtain the probability of recurrence calculated by the equation P = e^Logit(P)^/1 + e^Logit(P)^. Accordingly, *P* value could be calculated for every patient using corresponding clinicopathological parameters.

## 3 RESULTS

### 3.1 Study population

Among 1269 consecutive GC patients who had undergone curative resection, continuous and complete follow‐up data were recorded from 776 patients until 31 Jan 2018 (493 Patients followed up by local physicians). A total of 300 (38.7%) patients developed any patterns of recurrence (median time to recurrence was 12 months; range 2 to 60 months). Demographic and disease‐related features were shown in Table 1.

**Table 1:** Patient characteristics of patients with or without recurrence

Characteristics	Recurrence (No = 300, 38.7%)	No recurrence (No = 476, 61.3%)
Age		
Median	61	62
Range	22 ~ 88	26 ~ 90
≤60	146 (39.6%)	223 (60.4%)
>60	154 (37.8%)	253 (62.2%)
Gender		
Male	222 (40.7%)	323 (59.3%)
Female	78 (33.8%)	153 (66.2%)
Location of primary tumor		
Lower third	148 (37.5%)	247 (62.5%)
Middle third	79 (34.6%)	149 (65.4%)
Upper third	60 (50.8%)	58 (49.2%)
Gastroesophageal junction	6 (31.6%)	13 (68.4%)
More than 2/3 of stomach	7 (43.8%)	9 (56.2%)
Histology		
Well differentiated	6 (14.3%)	36 (85.7%)
Moderately differentiated	69 (34.2%)	133 (65.8%)
Poorly differentiated	161 (39.8%)	244 (60.2%)
Signet ring cell carcinoma	37 (50.0%)	37 (50.0%)
Mucinous adenocarcinoma	13 (48.1%)	14 (51.2%)
others	14 (53.8%)	12 (46.2%)
Maximum diameter (cm)		
Median	5.0	3.0
Range	0.5 ~ 15	0.1‐15
≤3	66 (21.0%)	249 (79.0%)
3.1‐6	181 (52.8%)	162 (47.2%)
>6	53 (44.9%)	65 (55.1%)
Perineural invasion		
Yes	163 (53.3%)	143 (46.7%)
No	137 (29.1%)	333 (70.9%)
Lymphovascular invasion		
Yes	181 (50.6%)	177 (49.4%)
No	119 (28.5%)	299 (71.5%)
Tumor stage		
Stage I	17 (7.1%)	222 (92.9%)
Stage II	63 (33.0%)	128 (67.0%)
Stage III	220 (63.6%)	126 (36.4%)
No. of positive lymph nodes		
Median	8	0
Range	0~52	0 ~ 50
No. of dissected lymph nodes		
Median	26	24
Range	15 ~ 76	15 ~ 92
Positive lymph node ratio		
Median	0.31	0.02
Range	0 ~ 1.0	0 ~ 1.0
Type of resection		
Subtotal gastrectomy	144 (31.6%)	312 (68.4%)
Total gastrectomy	156 (48.8%)	164 (51.2%)
Type of reconstruction		
Billroth I	51 (25.5%)	149 (74.5%)
Billroth II	88 (35.8%)	158 (64.2%)
Roux‐en‐Y	161 (48.8%)	169 (51.2%)
Adjuvant chemotherapy		
Yes	208 (46.7%)	237 (53.3%)
No	92 (27.8%)	239 (72.2%)

The median age of the 300 patients with recurrence at the time of gastrectomy was 61 years (range, 22 to 88 years). The incidence of recurrence for male patients was 222/545 or 40.7%. The most frequent primary tumor location was the lower third of the stomach and accounted for 148 patients. For patients initially diagnosed with perineural and lymphovascular invasion, the incidences of recurrence were 53.3% (n = 163) and 50.6% (n = 181), respectively. On the contrary, if patients had no perineural or lymphovascular invasion, recurrence occurred in 137 (29.1%) and 119 (28.5%) patients, respectively. Among the 300 cases with recurrences, overall tumor stage distribution included stage I (n = 17), II (n = 63), and III (n = 220). The median number of resected LNs and positive LNs was 26 and 8, respectively. Meanwhile, among the 300 patients who suffered recurrence, the number of patients who underwent a subtotal gastrectomy and a total gastrectomy was 144 and 156, respectively. Patients who received adjuvant chemotherapy had trends toward a higher incidence of recurrence comparing with those who did not receive chemotherapy after radical surgery, accounting for 46.7% (208 patients) and 27.8% (92 patients), respectively.

### 3.2 Overall recurrence patterns

The recurrence patterns among 776 patients who had complete follow‐up data were shown in Table 2. In all for the recurrence, there were 6.6% (n = 51) peritoneal recurrence, 19.4% (n = 151) regional recurrence, 7.2% (n = 56) local recurrence, and 21.1% (n = 164) distant recurrence. The site relatively prone to local recurrence was the anastomosis site (6.2%), followed by the gastric bed (1.4%). The highest incidence of distant recurrence included distant lymph node metastases (n = 66, 8.5%), liver (n = 58, 7.5%), and abdominal/pelvic wall (n = 30, 3.9%). Further details of distant organ and distant lymph node recurrences were listed in Table S1.

**Table 2:** Patterns of recurrence among 776 gastric cancer patients who had continuous and complete follow‐up data

	No. of patients (Percent %, n = 776)
PR	51 (6.6%)
Peritoneum	25 (3.2%)
Colorectum	25 (3.2%)
Ovary	6 (0.8%)
RR, regional lymph node	151 (19.4%)
LR	56 (7.2%)
Anastomotic site	48 (6.2%)
Gastric bed	11 (1.4%)
DR	164 (21.1%)
Liver	58 (7.5%)
Abdominal/pelvic wall	30 (3.9%)
Lung	10 (1.3%)
Bone	8 (1.0%)
Adrenal gland	7(0.9%)
Distant lymph node	66 (8.5%)
Pelvic nodes	46 (5.9%)
Other distant lymph nodes	20 (2.6%)
Other organs	17 (2.2%)

*Abbreviations:* DR, distant recurrence; LR, local recurrence; PR, peritoneal recurrence; RR, regional recurrence.

**Figure 1:**
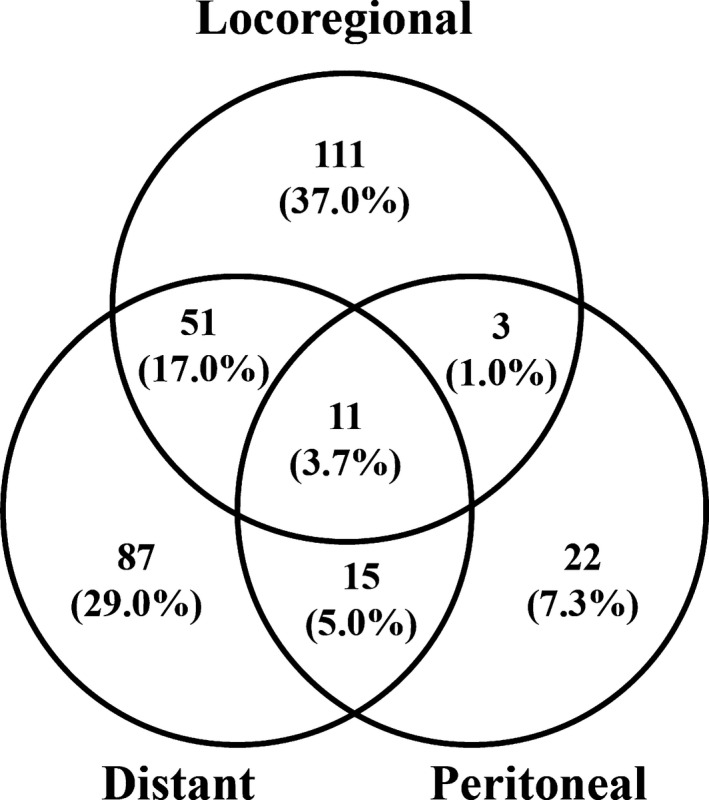
Patterns of recurrence in 300 patients after curative resection. Values in parentheses are percentages

The main patterns of recurrence in 300 patients were showed in Figure 1. For these patients with recurrence, 220 patients (73.3%) had recurrences involving only a single area; 69 patients (23.0%) had recurrences involving 2 areas, and the remaining 11 patients (3.7%) had recurrences involving all 3 areas. As a single pattern, locoregional recurrence (111 patients, 37.3%) was most frequent, followed by distant recurrence (87 patients, 29.0%) and peritoneal recurrence (22 patients, 7.3%).

### 3.3 Regional patterns of recurrence

Overall, as mentioned, among 776 patients who had complete follow‐up data, 151 patients suffered from regional recurrence. The most common sites of regional recurrence were paraaortic lymph node station 16a2 (LNs around the abdominal aorta, from the upper margin of the celiac trunk to the lower margin of the left renal vein, 11.0%), station 8 (LNs along the common hepatic artery, 9.5%), station 12 (LNs in the hepatoduodenal ligament, 6.7%), paraaortic lymph node station 16b1 (LNs around the abdominal aorta, from the lower margin of the left renal vein to the upper margin of the inferior mesenteric artery, 6.1%), and station 9 (LNs along the celiac artery, 5.0%) (Table 3). Regional recurrence was most seen outside the D2 dissected field, with 118 of 151 patients (78.1%) had involvement outside the D2 dissected field. In other words, 21.9% (33 of 151) patients had recurrence limited to nodal stations 1‐12 (data not shown).

**Table 3:** Rate (%) of lymph node metastases according to the different locations of patients with regional recurrence

Station No.	Node location	Total (n = 776)	Lower third (n = 395)	Middle third (n = 228)	Upper third (n = 118)	Gastroesophageal junction (n = 19)	More than 2/3 of stomach (n = 16)
1	Right paracardium	1.2 (9)	1.0 (4)	0.9 (2)	1.7 (2)	5.3 (1)	0
2	Left paracardium	0.9 (7)	0.3 (1)	0.9 (2)	2.5 (3)	5.3 (1)	0
3	Along the lesser curvature	1.9 (15)	1.8 (7)	1.8 (4)	1.7 (2)	5.3 (1)	6.3 (1)
4	Along the great curvature	0.9 (7)	0.8 (3)	0.4 (1)	1.7 (2)	5.3 (1)	0
5	Suprapylorum	1.2 (9)	0.8 (3)	1.3 (3)	1.7 (2)	5.3 (1)	0
6	Infrapylorum	1.7 (13)	1.8 (7)	1.3 (3)	1.7 (2)	5.3 (1)	0
7	Along the left gastric artery	2.7 (21)	2.5 (10)	2.2 (5)	3.4 (4)	5.3 (1)	6.3 (1)
8	Along the common hepatic artery	9.5 (74)	9.6 (38)	8.3 (19)	11.0 (13)	15.8 (3)	6.3 (1)
9	Around the celiac artery	5.0 (39)	4.3 (17)	4.4 (10)	7.6 (9)	10.5 (2)	6.3 (1)
10	At the splenic hilum	1.3 (10)	0.8 (3)	0.9 (2)	3.4 (4)	5.3 (1)	0
11	Along the proximal splenic artery	3.6 (28)	2.3 (9)	5.3 (12)	4.2 (5)	5.3 (1)	6.3 (1)
12	In the hepatoduodenal ligament	6.7 (52)	7.1 (28)	4.8 (11)	5.9 (7)	21.1 (4)	12.5 (2)
13	On the posterior surface of the pancreatic head	4.5 (35)	4.3 (17)	4.8 (11)	3.4 (4)	10.5 (2)	6.3 (1)
14	Along the superior mesenteric vein/artery	3.5 (27)	3.0 (12)	3.5 (8)	3.4 (4)	5.3 (1)	12.5 (2)
15	Along the middle colic vessels	1.0 (8)	1.0 (4)	0.9 (2)	0.9 (1)	5.3 (1)	0
16a1	Around the abdominal aorta	3.1 (24)	2.8 (11)	1.8 (4)	5.9 (7)	5.3 (1)	6.3 (1)
16a2	Around the abdominal aorta	11.0 (85)	11.4 (45)	7.5 (17)	16.1 (19)	10.5 (2)	12.5 (2)
16b1	Around the abdominal aorta	6.1 (47)	6.1 (24)	4.4 (10)	8.5 (10)	10.5 (2)	6.3 (1)
16b2	Around the abdominal aorta	2.7 (21)	3.0 (12)	1.8 (4)	2.5 (3)	5.3 (1)	6.3 (1)

Note

Numbers in parentheses indicate the number of patients with positive lymph nodes.

Table 3 also showed the prevalence and rate of metastatic LNs in first regional recurrence among 776 patients who had complete follow‐up data according to different tumor locations. For the distal third of the stomach, the most common sites of regional recurrence were station 16a2 (11.4%), station 8 (9.6%), station 12 (7.1%), and station 16b1 (6.1%); station 8 (8.3%), station 16a2 (7.5%), and station 11 (LNs along the splenic artery, 5.3%) for the middle third; and station 16a2 (16.1%), station 8 (11.0%), station 16b1 (8.5%), station 9 (7.6%), station 12 (5.9%), and station 16a1 (paraaortic LNs in the diaphragmatic aortic hiatus, 5.9%) for the proximal third. For tumors located in the gastroesophageal junction and more than two third of stomach, total sample size was too limited to draw definite conclusions.

### 3.4 Clinicopathological characteristics and risk prediction models for recurrence

**Figure 2:**
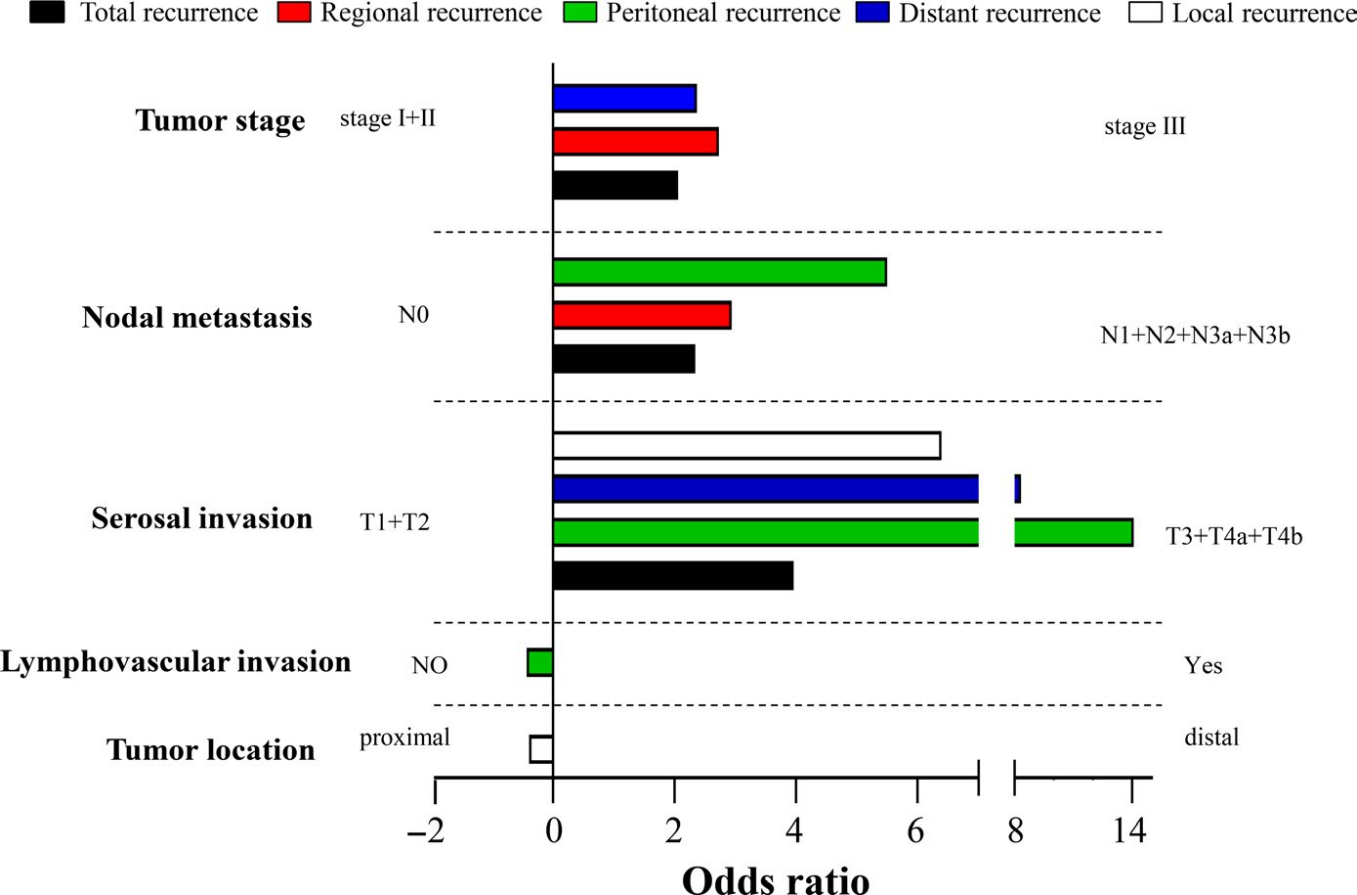
Recurrence risk factors according to recurrent pattern by multivariate analysis (*P *＜ 0.05)

Table S2 demonstrated univariate and multivariate logistic regression analysis of independent risk factors for prediction of first recurrence patterns (patient characteristics according to different recurrence patterns were listed in Table S3). Clinicopathological characteristics, including receiving adjuvant chemotherapy, type of reconstruction (Roux‐en‐Y), type of resection (total gastrectomy), overall tumor stage, lymphovascular invasion, nerve invasion, maximum diameter (greater than 3 centimeters), histology (undifferentiated of tumor), and proximally located tumor were found to be correlated with recurrence risk of GC patients. A further multivariate analysis (Figure 2) showed that T stage, node metastasis, and overall tumor stage had significant impact on total recurrence. In addition, for specific patterns of recurrence, regional recurrence was closely associated with nodal metastasis (N1+N2+N3a+N3b) and tumor stage; peritoneal recurrence was closely associated with nodal metastasis, serosal invasion (T3+T4a+T4b), and without lymphovascular invasion; distant recurrence was closely associated with serosal invasion and tumor stage; local recurrence was closely associated with serosal invasion and proximal tumor location.

Median time to recurrence for total patient group was 12 months, and the median recurrence time for peritoneal recurrence, regional recurrence, local recurrence, and distant recurrence was 10, 10, 15.5, and 10 months, respectively. Patients were next divided into an early recurrence group (n = 159, within 12 months after primary gastrectomy) and a late recurrence group (n = 141, exceeding 12 months after primary gastrectomy) for correlation with clinic‐pathologic features (Table 4). Clinic‐pathologic features, including lymphovascular invasion, lymph node metastasis, and tumor stage, were closely related to early recurrence.

**Table 4:** Clinicopathologic features according to recurrence time

	Early recurrence (≤12 months)	Late recurrence (>12 months)	*P* value
Numbers	159	141	
Median age (years)	61	60	0.310
Gender			0.132
Male	115	107	
Female	44	34	
Location of primary tumor			0.571
Lower third	77	71	
Middle third	45	34	
Upper third	28	32	
Gastroesophageal junction	4	2	
More than 2/3 of stomach	5	2	
Histology			0.255
Well differentiated	2	4	
Moderately differentiated	33	36	
Poorly differentiated	94	67	
Signet ring cell carcinoma	20	17	
Mucinous adencarcinoma	5	8	
others	5	9	
Maximum diameter (cm)			0.087
≤3	27	39	
3.1‐6	98	83	
>6	34	19	
Median	5	4.5	
Nerve invasion			0.418
Yes	90	73	
No	69	68	
Lymphovascular invasion			<0.001
Yes	113	68	
No	46	73	
Tumor stage			<0.001
Stage I	3	14	
Stage II	26	37	
Stage III	130	90	
No. of positive lymph nodes			<0.001
Median	10	5	
No. of dissected lymph nodes			0.320
Median	27	25	
Lymph node ratio			<0.001
Median	0.4	0.23	
Type of resection			0.165
Subtotal gastrectomy	70	74	
Total gastrectomy	89	67	
Type of reconstruction			0.107
Billroth I	21	30	
Billroth II	46	42	
Roux‐en‐Y	92	69	
Adjuvant chemotherapy			0.386
Yes	109	99	
No	50	42	

**Figure 3:**
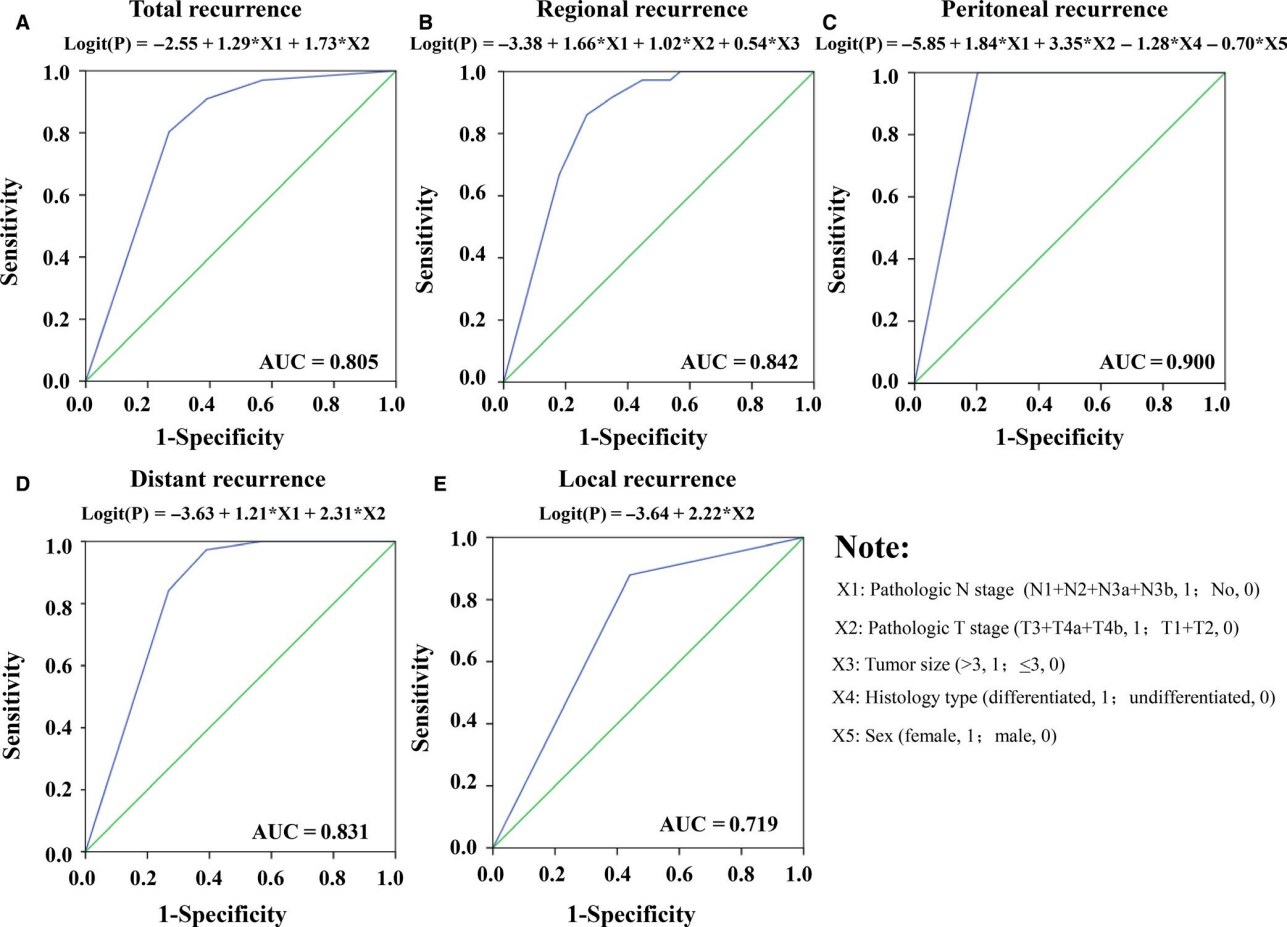
Receiver operating characteristic (ROC) curves analyzed the accuracy of the models’ ability to predict the probability of recurrence based on clinicopathological features: (a) total recurrence; (b) regional recurrence; (c) peritoneal recurrence; (d) distant recurrence; and (e) local recurrence. The probability of recurrence can be calculated further by the equation *P* = e^Logit(P)^/1 + e^Logit(P)^. AUC: area under the ROC curve

Figure 3 revealed the receiver operating characteristic (ROC) curves of different prognostic models for prediction of recurrence according to the recurrence patterns. The final model was based upon five independent variables, namely, X1 (pathologic N stage), X2 (pathologic T stage), X3 (tumor size), X4 (histology differentiation degree), and X5 (gender). These related clinic‐pathological characteristics could be utilized together to get the area under the ROC curves (AUCs) of 0.805, 0.842, 0.900, 0.831, and 0.719, respectively, for total, regional, peritoneal, distant, and local recurrence. As shown in Figure 3, the *P* value (probability of recurrence) could be calculated by the equation *P* = e^Logit(P)^/1 + e^Logit(P)^ for every patient using corresponding clinical and pathological parameters.

## 4 DISCUSSION

Several studies have attempted to determine patterns of GC recurrence with variable results. The disagreements of published data might be due to the selection in patient cohorts, the time points at which recurrence were determined, the criteria by which recurrence patterns were classified, and the different extent of surgical resection, especially the extent of lymph node dissection.^14^ In this study, the site most likely to recur was distant recurrence (164 patients), comprising 21.1% of all recurrences after D2 gastrectomy. Even higher distant recurrence rates in other institutions (varying from 37% to 46.3%) supported the notion that hematogenous metastasis should be monitored carefully after radial dissection.^15^ Lower incidence of peritoneal metastases was found in the current series (n = 51, 6.6%) versus the Japan Clinical Oncology Group (JCOG9501) trial (among all recurrence pattern, the most frequent recurrence site was the peritoneum, which accounted for 38.1% of all 215 patients with recurrences and 15.7% of a total of 523 patients) and a recent Swedish study (peritoneal seeding was found in almost half of the GC patients after a curative gastrectomy).^16^, ^17^ It might be due to relatively insensitive imaging examination (even magnetic resonance imaging with only 56% sensitivity for peritoneum implanting)^18^ and less aggressive peritoneal evaluation by laparoscopic exploration in the current series.

Understanding the incidence and location of local‐regional recurrence was imperative for proper conceptualization of RT treatment planning and minimize radiation‐related toxicity.^19^ In our present study, 56 (7.2%) patients suffered from local recurrence. The site relatively prone to recur was the anastomosis site (6.2%), which should be included in adjuvant RT field considering the high recurrence risk. One of the major variations in RT fields was the remnant stomach, which was not included in the ARTIST trial while was included in the INT‐0116 trial.^20^ GC patients who were treated with subtotal gastrectomy and adjuvant CRT were retrospectively analyzed. No significant differences were found for survival rates whether the remnant stomach was included in the RT field or not.^20^ In our present study, no remnant stomach recurrence was found. In general, it seemed reasonable to exclude the remnant stomach from adjuvant RT field for patients with negative surgical/pathologic margins. Gastric bed recurrence was also not prominent (1.4%). Since the incidence of gastric bed recurrence was accurate only in reoperative and autopsy analyses, it was inaccurately low in most if not all clinical series. Currently, gastric bed was included in the target volume if there was any evidence of adjacent organs invasion. Besides, if all nodal groups at 5% or higher risk, by primary site, were included in an irradiation field, the gastric bed (head/body of pancreas varies by site of primary lesion) would essentially be in most fields and should not be purposely excluded. However, GC was not routinely managed by complete D2 resection in North American or European centers, the patterns of recurrence in this study might not apply to D0/D1 dissection. Gastric bed and remnant stomach were still recommended to be included in the RT target volume for D0/D1 dissection.

In this study, 151 of the patients (19.4%) suffered from regional recurrence, which was a high incidence rate.^21^ Different patterns of regional recurrence were investigated according to primary tumor site. If lymph node metastases exceeding 5%, an empirical cutoff value, was regarded as high risk, the nodal regions recommended to be covered in an irradiation field were as follows: station 16a2 (11.4%), station 8 (9.6%), station 12 (7.1%), and station 16b1 (6.1%) for the distal third of the stomach; station 8 (8.3%), station 16a2 (7.5%), and station 11 (LNs along the splenic artery, 5.3%) for the middle third; and station 16a2 (16.1%), station 8 (11.0%), station 16b1 (8.5%), station 9 (7.6%), station 12 (5.9%), and station 16a1 (5.9%) for the proximal third. Overall, among patients with regional recurrence, the most common sites were paraaortic lymph node station 16a2 (11.0%), station 8 (9.5%), station 12 (6.7%), paraaortic lymph node station 16b1 (6.1%), and station 9 (5.0%). For patients receiving standard D2 lymphadenectomy performed by well‐trained surgeons, exclusion of perigastric LNs in the RT field to reduce toxicity might be considered. Paraaortic LNs and its main branches seemed the most common site of recurrence for GC. In addition, among the patients with regional recurrence to lymph node stations 16a2 and 16b1, patients were further categorized as 16a2‐only group when tumors metastasized to only the lymph node station 16a2, as 16a2 + 16b1 group if tumors metastasized to both the stations 16a2 and 16b1, and as a skip group if metastatic LNs were only in station 16b1. The numbers of cases in 16a2‐only group, 16a2 + 16b1 group, and skip group were 48, 37, and 10 respectively. As station 16b1 rarely relapsed isolatedly, station 16b1 metastases might be prevented from the proposed radiation volume if proximal stations were under well control. In addition, regional recurrence was most seen outside the D2 dissected field, 21.9% (33 of 151) patients had recurrence limited to nodal stations 1‐12. Yoon et al analyzed follow‐up images from 91 patients to determine first regional recurrence after D2 lymphadenectomy in stage III GC with N3 disease. The most commonly involved first recurrence LNs were No. 16b and No. 16a.^22^ A Korea study suggested that the most prevalent lymph node recurrences were in stations Nos. 9, 12, 13, 14, and No.16, which were mainly located outside the D2 dissection field.^23^ Yu et al investigated optimal RT target volume from the results of the Adjuvant Chemoradiation Therapy in Stomach Cancer (ARTIST) trial, and found that the LNs in groups 2 and 3 including the paraaortic, retropancreatic, aortocaval, and retrocaval region might be the most important RT target.^24^ Therefore, optimizing regional control of LNs outside the D2 dissected area (stations 13‐16) was crucial for rational design of the RT field. Additional large prospective studies were required to guide tailored irradiation of LNs stations and evaluate the optimal individualized target volume.

Related clinicopathological elements of GC patients should also be considered comprehensively by radiation oncologists to customize the radiation field and predict prognosis. In our study, patients with risk factors, such as tumor maximum diameter greater than 3 centimeters, undifferentiated histology, advanced tumor stage, and nerve and lymphovascular invasion could be candidates for adjuvant CRT. Clinicopathological characteristics, including receiving total gastrectomy and adjuvant chemotherapy, were also associated with the recurrence risk of GC patients. It might be due to the limitation of a retrospective study, and for a patient who received total gastrectomy and adjuvant chemotherapy, there was a tendency of more advanced disease. Similarly, Mehmedagic et al found that TNM stage and tumor histological type had a significant value for the GC recurrence.^25^ In another study, pattern of LNs metastases was correlated with the maximum tumor diameter, T stage, macroscopic types, and histologic differentiation.^26^ Nevertheless, little literature was available regarding time to recurrence. Patients were dichotomized further by recurrence time using cut‐off value of 1 year.^27^ If a tumor recurred within the first year after radical resection, it was likely to be aggressive and to be related with a dismal prognosis. Independent risk factors for early recurrence were tumors with lymphovascular invasion, nodal metastases, and advanced tumor stage. The timing of recurrence seemed to be affected by tumor aggressiveness rather than treatment modalities, such as adjuvant chemotherapy, type of resection, and reconstruction. It was suggested that early recurrence indicated a dismal prognosis and death risk was twofold higher than those GC patients with late recurrence.^28^ These early recurrence‐related clinicopathological features might be available for predicting prognosis of GC patients.

These recurrence‐related variables were finally entered into logistic regression to establish recurrence risk prediction models. Our new model could well predict the probability of recurrence for GC patients who have undergone curative resection and might potentially be useful in future clinical practice to determine which patients should receive postoperative CRT. The identified independent risk factors of recurrence and the recurrence prediction model need to be further validated in an independent population.^29^

Our study analyzed a relatively large sample of GC patients retrospectively and patterns, incidences, and time of recurrence were illustrated. All the detailed information was critical for clinical monitoring patients of appropriate therapeutic modalities and individualized target coverage for adjuvant RT. The new logistic regression model might act as a useful tool to evaluate recurrence risk and determine which patients should receive postoperative CRT. Although the potential selection biases inherent to a retrospective study, sample size limitations, and missing data, the data presented are of a practical nature and have the potential to inform RT field design. These findings offered important information in clinical monitoring patients of appropriate therapeutic modalities and supplying the opportunity for individualized target coverage. Larger multiple‐center prospective studies are required to determine the role of adjuvant RT in resected GC and assess the individualized radiation fields.

## AUTHOR CONTRIBUTIONS

**Jing Xu:** Wrote the article and analysis of data and statistics. **Li Shen:** Reviewed and edited the article. **Yongjie Shui:** History review. **Wei Yu:** Developed the logistic regression models. **Qingqu Guo:** Analysis of data and statistics. **Risheng Yu:** Image evaluation. **Yulian Wu:** Reviewed and edited the article. **Qichun Wei:** Designed, revised, and supervised the writing and concept of the article.

## Supporting information

Table S1

Table S2

Table S3

## Data Availability

The data will be provided upon request.
