# Supplementary material for: Patterns of recurrence after curative D2 resection for gastric cancer: Implications for postoperative radiotherapy
Source: Cancer Med. 2020 May 18;9(13):4724–35. doi: 10.1002/cam4.3085 (PMC7333831; doi:10.1002/cam4.3085)
Supplement: Supplementary file 1 — Table S1 [file CAM4-9-4724-s001.docx]

**Table E1. Details of distant recurrence among 776 gastric cancer patients who had continuous and complete follow-up data.**

|  | **No. of patients (Percent %, n=776)** |  |
| --- | --- | --- |
| **Distant recurrence** | 164 (21.1%) |  |
| **Liver** | 58 (7.5%) |  |
| **Abdominal/pelvic wall** | 30 (3.9%) |  |
| **Lung** | 10 (1.3%) |  |
| **Bone** | 8 (1.0%) |  |
| **Adrenal gland** | 7 (0.9%) |  |
| **Pancreas** | 5 |  |
| **Brain** | 3 |  |
| **Pleura** | 3 |  |
| **Duodenum** | 2 |  |
| **Kidney** | 1 |  |
| **Gall bladder** | 1 |  |
| **Epityphlon** | 1 |  |
| **Ileum** | 1 |  |
| **Distant lymph node** | 66 (8.5%) |  |
| **Pelvic nodes** | 46 (5.9%) |  |
| **Supra- and infraclavicula nodes** | 6 (0.8%) |  |
| **Cardiodiaphragmatic angle nodes** | 5 |  |
| **Inguinal nodes** | 4 |  |
| **Pulmonary and mediastinal nodes** | 4 |  |
| **Axillary nodes** | 1 |  |
