# Supplementary material for: Patterns of recurrence after curative D2 resection for gastric cancer: Implications for postoperative radiotherapy
Source: Cancer Med. 2020 May 18;9(13):4724–35. doi: 10.1002/cam4.3085 (PMC7333831; doi:10.1002/cam4.3085)
Supplement: Supplementary file 2 — Table S2 [file CAM4-9-4724-s002.docx]

**Table E2. Univariate and multivariate analysis of clinicopathologic factors potentially associated with presence of first recurrence pattern (significant differences are marked with bold font).**

| **Factor** | **Recurrence types** | **Univariate analysis** | | |  | **Multivariate analysis** | | |
| --- | --- | --- | --- | --- | --- | --- | --- | --- |
|  |  | β | P | Odds ratio |  | β | P | Odds ratio |
| **Age** (>60; <60) | Total | -0.07 | 0.62 | 0.93 (0.70-1.24) |  | -0.15 | 0.41 | 0.86 (0.60-1.23) |
|  | Regional | -0.01 | 0.97 | 0.99 (0.69-1.43) |  | -0.15 | 0.51 | 0.86 (0.55-1.35) |
|  | Peritoneal | **-0.65** | **0.03** | **0.52 (0.29-0.95)** |  | -0.53 | 0.17 | 0.59 (0.28-1.26) |
|  | Distant | 0.13 | 0.47 | 1.14 (0.80-1.63) |  | -0.04 | 0.85 | 0.96 (0.61-1.50) |
|  | Local | -0.13 | 0.65 | 0.88 (0.51-1.52) |  | -0.34 | 0.29 | 0.71 (0.38-1.33) |
|  |  |  |  |  |  |  |  |  |
| **Sex**  (male, female) | Total | -0.30 | 0.07 | 0.74 (0.54-1.02) |  | -0.14 | 0.49 | 0.87 (0.59-1.29) |
|  | Regional | **-0.61** | **0.01** | **0.55 (0.35-0.85)** |  | -0.43 | 0.10 | 0.65 (0.39-1.09) |
|  | Peritoneal | 0.39 | 0.19 | 0.52 (0.29-0.95) |  | 0.51 | 0.17 | 1.66 (0.80-3.43) |
|  | Distant | -0.31 | 0.13 | 0.73 (0.49-1.09) |  | -0.01 | 0.97 | 0.99 (0.61-1.61) |
|  | Local | -0.31 | 0.32 | 0.74 (0.40-1.37) |  | -0.12 | 0.72 | 0.88 (0.45-1.74) |
|  |  |  |  |  |  |  |  |  |
| **Location of primary tumor** (distal, proximal) | Total | **-0.47** | **0.01** | **0.63 (0.44-0.90)** |  | -0.07 | 0.77 | 0.93 (0.58-1.50) |
|  | Regional | -0.33 | 0.16 | 0.72 (0.46-1.14) |  | 0.17 | 0.58 | 1.18 (0.65-2.13) |
|  | Peritoneal | -0.31 | 0.39 | 0.74 (0.36-1.49) |  | -0.05 | 0.91 | 0.95 (0.38-2.37) |
|  | Distant | **-0.57** | **0.01** | **0.56 (0.37-0.86)** |  | -0.14 | 0.63 | 0.87 (0.50-1.52) |
|  | Local | **-0.88** | **0.00** | **0.42 (0.23-0.76)** |  | **-0.93** | **0.02** | **0.39 (0.18-0.86)** |
|  |  |  |  |  |  |  |  |  |
| **Histology** (differentiated, undifferentiated) | Total | **-0.50** | **0.00** | **0.61 (0.44-0.84)** |  | -0.07 | 0.75 | 0.94 (0.63-1.39) |
|  | Regional | **-0.60** | **0.00** | **0.55 (0.36-0.84)** |  | -0.23 | 0.38 | 0.79 (0.49-1.31) |
|  | Peritoneal | **-1.87** | **0.00** | **0.16 (0.06-0.44)** |  | -1.03 | 0.07 | 0.36 (0.12-1.10) |
|  | Distant | -0.37 | 0.07 | 0.69 (0.47-1.03) |  | 0.16 | 0.52 | 1.17 (0.72-1.90) |
|  | Local | **-0.75** | **0.03** | **0.47 (0.24-0.92)** |  | -0.54 | 0.14 | 0.59 (0.29-1.20) |
|  |  |  |  |  |  |  |  |  |
| **Maximum diameter** (>3.0, <3.0) | Total | **1.36** | **0.00** | **3.89 (2.80-5.39)** |  | 0.326 | 0.12 | 1.39 (0.92-2.08) |
|  | Regional | **1.53** | **0.00** | **4.62 (2.96-7.19)** |  | 0.49 | 0.07 | 1.63 (0.96-2.78) |
|  | Peritoneal | **1.17** | **0.00** | **3.21 (1.67-6.17)** |  | -0.08 | 0.86 | 0.93 (0.41-2.09) |
|  | Distant | **1.35** | **0.00** | **3.87 (2.56-5.89)** |  | 0.20 | 0.44 | 1.22 (0.73-2.04) |
|  | Local | **1.44** | **0.00** | **4.21 (2.17-8.14)** |  | 0.59 | 0.14 | 1.80 (0.83-3.90) |
|  |  |  |  |  |  |  |  |  |
| **Nerve invasion** (Yes, No) | Total | **1.02** | **0.00** | **2.77 (2.05-3.74)** |  | -0.08 | 0.68 | 0.92 (0.62-1.36) |
|  | Regional | **0.94** | **0.00** | **2.56 (1.76-3.72)** |  | -0.28 | 0.26 | 0.76 (0.46-1.23) |
|  | Peritoneal | **1.72** | **0.00** | **5.59 (2.97-10.5)** |  | 0.59 | 0.15 | 1.80 (0.81-4.00) |
|  | Distant | **1.00** | **0.00** | **2.73 (1.90-3.94)** |  | -0.20 | 0.40 | 0.82 (0.52-1.31) |
|  | Local | **0.98** | **0.00** | **2.67 (1.54-4.64)** |  | -0.09 | 0.80 | 0.92 (0.47-1.80) |
|  |  |  |  |  |  |  |  |  |
| **Lymphovascular invasion** (Yes, No) | Total | **0.94** | **0.00** | **2.57 (1.91-3.46)** |  | -0.12 | 0.55 | 0.89 (0.60-1.32) |
|  | Regional | **1.20** | **0.00** | **3.31 (2.25-4.87)** |  | 0.24 | 0.33 | 1.28 (0.78-2.09) |
|  | Peritoneal | **0.64** | **0.03** | **1.90 (1.06-3.40)** |  | **-0.85** | **0.03** | **0.43 (0.20-0.92)** |
|  | Distant | **0.99** | **0.00** | **2.68 (1.86-3.87)** |  | -0.11 | 0.64 | 0.89 (0.55-1.44) |
|  | Local | **0.87** | **0.00** | **2.39 (1.37-4.17)** |  | 0.12 | 0.74 | 1.13 (0.57-2.23) |
|  |  |  |  |  |  |  |  |  |
| **Overall stage** (I-II, III) | Total | **2.03** | **0.00** | **7.64 (5.51-10.6)** |  | **0.72** | **0.00** | **2.06 (1.24-3.42)** |
|  | Regional | **2.22** | **0.00** | **9.21 (5.99-14.1)** |  | **1.02** | **0.00** | **2.73 (1.42-5.22)** |
|  | Peritoneal | **2.43** | **0.00** | **11.4 (5.54-23.4)** |  | 0.60 | 0.22 | 1.83 (0.70-4.76) |
|  | Distant | **2.21** | **0.00** | **9.14 (6.03-13.9)** |  | **0.86** | **0.01** | **2.37 (1.24-4.52)** |
|  | Local | **1.37** | **0.00** | **3.94 (2.25-6.90)** |  | 0.49 | 0.30 | 1.63 (0.65-4.01) |
|  |  |  |  |  |  |  |  |  |
| **Type of resection** (Total, Subtotal) | Total | **0.72** | **0.00** | **2.06 (1.53-2.77)** |  | 0.12 | 0.76 | 1.13 (0.52-2.43) |
|  | Regional | **0.66** | **0.00** | **1.93 (1.33-2.79)** |  | -0.04 | 0.94 | 0.96 (0.36-2.58) |
|  | Peritoneal | **0.92** | **0.00** | **2.51 (1.40-4.50)** |  | 1.03 | 0.16 | 2.80 (0.67-11.7) |
|  | Distant | **0.85** | **0.00** | **2.35 (1.63-3.37)** |  | 0.31 | 0.55 | 1.37 (0.49-3.80) |
|  | Local | 0.51 | 0.07 | 1.66 (0.96-2.87) |  | -0.15 | 0.85 | 0.86 (0.18-4.13) |
|  |  |  |  |  |  |  |  |  |
| **Surgical reconstruction** (Roux-en-Y, Billroth-I+Billroth-II) | Total | **0.74** | **0.00** | **2.10 (1.57-2.83)** |  | 0.054 | 0.89 | 1.06 (0.49-2.25) |
|  | Regional | **0.72** | **0.00** | **2.05 (1.41-2.97)** |  | 0.31 | 0.54 | 1.36 (0.51-3.62) |
|  | Peritoneal | **0.72** | **0.02** | **2.04 (1.14-3.65)** |  | -0.93 | 0.20 | 0.39 (0.09-1.65) |
|  | Distant | **0.88** | **0.00** | **2.41 (1.68-3.47)** |  | -0.05 | 0.91 | 0.95 (0.34-2.66) |
|  | Local | 0.53 | 0.06 | 1.70 (0.98-2.93) |  | -0.30 | 0.71 | 0.74 (0.15-3.58) |
|  |  |  |  |  |  |  |  |  |
| **Adjuvant chemotherapy** (Yes, No) | Total | **0.90** | **0.00** | **2.47 (1.82-3.36)** |  | -0.16 | 0.43 | 0.85 (0.57-1.27) |
|  | Regional | **0.90** | **0.00** | **2.45 (1.65-3.64)** |  | -0.19 | 0.46 | 0.83 (0.50-1.37) |
|  | Peritoneal | **1.08** | **0.00** | **2.95 (1.53-5.67)** |  | -0.60 | 0.15 | 0.55 (0.24-1.25) |
|  | Distant | **0.82** | **0.00** | **2.28 (1.56-3.33)** |  | -0.42 | 0.09 | 0.66 (0.40-1.07) |
|  | Local | **0.73** | **0.01** | **2.07 (1.16-3.69)** |  | -0.32 | 0.36 | 0.73 (0.37-1.44) |
