# Supplementary material for: Patterns of recurrence after curative D2 resection for gastric cancer: Implications for postoperative radiotherapy
Source: Cancer Med. 2020 May 18;9(13):4724–35. doi: 10.1002/cam4.3085 (PMC7333831; doi:10.1002/cam4.3085)
Supplement: Supplementary file 3 — Table S3 [file CAM4-9-4724-s003.docx]

**Table E3. Patient characteristics according to different recurrence patterns of recurrence.**

|  | **PR** | **RR** | **LR** | **DR** |
| --- | --- | --- | --- | --- |
| **Number of patients** | 51 | 151 | 56 | 164 |
| **Median age (years)** | 57 | 62 | 61.5 | 62 |
| **Sex** |  |  |  |  |
| Male | 30 | 120 | 41 | 122 |
| Female | 21 | 31 | 15 | 42 |
| **Location of primary tumor** |  |  |  |  |
| Lower third | 24 | 84 | 27 | 77 |
| Middle third | 16 | 34 | 10 | 44 |
| Upper third | 7 | 27 | 18 | 32 |
| Gastroesophageal junction | 1 | 4 | 1 | 6 |
| More than 2/3 of stomach | 3 | 2 | 0 | 5 |
| **Histology** |  |  |  |  |
| Well differentiated | 2 | 3 | 2 | 3 |
| Moderately differentiated | 2 | 32 | 10 | 42 |
| Poorly differentiated | 31 | 83 | 32 | 82 |
| Signet ring cell carcinoma | 13 | 17 | 6 | 20 |
| Mucinous adencarcinoma | 3 | 9 | 4 | 6 |
| others | 0 | 7 | 2 | 11 |
| **Maximum diameter (cm)** |  |  |  |  |
| ≤3 | 12 | 29 | 12 | 36 |
| 3.1-6 | 25 | 99 | 35 | 99 |
| ＞6 | 14 | 23 | 9 | 29 |
| Median | 4.5 | 5 | 5 | 5 |
| **Nerve invasion** |  |  |  |  |
| Yes | 36 | 79 | 29 | 89 |
| No | 15 | 72 | 27 | 75 |
| **Lymphovascular invasion** |  |  |  |  |
| Yes | 27 | 100 | 32 | 101 |
| No | 24 | 51 | 24 | 63 |
| **Tumor stage** |  |  |  |  |
| Stage I | 0 | 11 | 6 | 7 |
| Stage II | 10 | 24 | 16 | 32 |
| Stage III | 41 | 116 | 34 | 125 |
| **No. of positive lymph nodes** |  |  |  |  |
| Median | 10 | 9 | 5.5 | 7 |
| **No. of dissected lymph nodes** |  |  |  |  |
| Median | 28 | 26 | 26 | 26 |
| **Positive Lymph node ratio** |  |  |  |  |
| Median | 0.39 | 0.38 | 0.24 | 0.29 |
| **Type of resection** |  |  |  |  |
| Subtotal gastrectomy | 22 | 75 | 29 | 74 |
| Total gastrectomy | 29 | 76 | 27 | 90 |
| **Type of reconstruction** |  |  |  |  |
| Billroth-I | 6 | 28 | 11 | 24 |
| Billroth-II | 18 | 43 | 18 | 47 |
| Roux-en-Y | 27 | 80 | 27 | 93 |
| **Adjuvant chemotherapy** |  |  |  |  |
| Yes | 40 | 108 | 40 | 120 |
| No | 11 | 43 | 16 | 44 |

***Abbreviations:* PR=peritoneal recurrence; RR=regional recurrence; LR=local recurrence; DR=distant recurrence.**
